# Circulating Endothelial Microparticles and Aortic Stiffness in Patients with Type 2 Diabetes Mellitus

**DOI:** 10.3390/medicina55090596

**Published:** 2019-09-16

**Authors:** Malgorzata Dec-Gilowska, Marcin Trojnar, Boguslaw Makaruk, Marcin Czop, Sylwia Przybylska-Kuc, Barbara Mosiewicz-Madejska, Grzegorz Dzida, Jerzy Mosiewicz

**Affiliations:** 1 Chair and Department of Internal Diseases, Medical University of Lublin, 20-059 Lublin, Poland; malgosdec@gmail.com (M.D.-G.); boguslawmakaruk@umlub.pl (B.M.); 1@wp.pl (S.P.-K.); grzegorzdzida@umlub.pl (G.D.); jerzy.mosiewicz@gmail.com (J.M.); 2 Chair and Department of Internal Diseases and Diabetology, Medical University of Warsaw, 02-091 Warsaw, Poland; 3 Department of Clinical Genetics, Medical University of Lublin, 20-059 Lublin, Poland; marcinczop@umlub.pl; 4 Chair and Department of Internal Diseases, Medical University of Lublin, Students Medical Association, 20-059 Lublin, Poland; bmosiewicz@gmail.com

**Keywords:** aortic stiffness, type 2 diabetes mellitus, elasticity index, compliance index

## Abstract

*Background and objectives:* Diabetes mellitus represents a metabolic disorder the incidence of which has been on the increase in recent years. The well-known long-term complications of this disease encompass a wide spectrum of renal, neurological and cardiovascular conditions. The aim of the study was to investigate the serum concentration of endothelial microparticles (EMPs) as well as selected noninvasive parameters of the ascending aorta stiffness calculated with echocardiography. *Materials and Methods:* 58 patients were enrolled in this study—38 subjects diagnosed with type 2 diabetes mellitus (T2DM) and 20 healthy controls. The analyzed populations did not differ significantly with respect to age, renal function, systolic and diastolic blood pressure. *Results:* The patients with T2DM and concomitant hypertension presented higher levels of EMPs in comparison with diabetic normotensive subjects. Among patients with T2DM and hypertension, aortic stiffness assessed with the elasticity index (Ep) was higher and the aortic compliance index (D) lower than in the diabetic normotensive group. No correlation between the amount of EMPs and lipid profile, C-reactive protein (CRP) level and glycemia, was observed in the studied group. There was, however, a statistically significant positive correlation between the creatinine level and amount of EMPs, while the negative relationship was documented for EMPs level and the estimated glomerular filtration rate (eGFR). *Conclusions:* Considering the elevated number of EMPs in diabetic patients with hypertension as well as the positive correlation between EMPs and serum creatinine level, EMPs assessment could be useful in identifying patients who are at high risk of organ damage due to diabetes mellitus.

## 1. Introduction

Diabetes mellitus is a metabolic disease characterized by chronic hyperglycemia and long-lasting disorders in the carbohydrate, lipid, and protein metabolism, which leads to chronic progressive dysfunction and failure of the nervous, renal and cardiovascular systems [1]. There are a great many of studies indicating an increase in the cardiovascular risk among patients with type 2 diabetes mellitus (T2DM) [2,3].

Vascular endothelium is an active tissue covering the interior surface of blood vessels and it constitutes an interface between circulating blood and other tissues of the body. The endothelium plays a crucial role in blood pressure regulation, angiogenesis and hemostasis by secreting various biologically active substances [4]. Chronic inflammation within the vessel wall and endothelial dysfunction are essential in the pathogenesis of atherosclerosis. The structural changes in the arteries lead to their stiffness. Previous studies emphasize that arterial stiffness is increased in the course of diabetes, which is believed to be an independent prognostic factor of death as well as cardiovascular death [5].

Endothelial microparticles (EMPs) are the cellular markers of endothelial dysfunction. EMPs are membrane vesicles derived from activated or apoptotic endothelial cells [6]. Their function has not been fully established. They take part in the inflammation, thrombosis and angiogenesis processes and may interfere with vascular hemostasis and contribute to atherosclerosis and its complications [7]. Arterial elasticity is an important parameter indicating the state of the blood vessel walls [8]. Endothelium damage and a decrease in arterial elasticity are connected to each other. Endothelial dysfunction promotes local atherosclerotic plaque formation and, at the same time, it decreases the ability of the vessels to dilate. Arterial stiffening causes an increase in pulse pressure, which leads to pulsating blood flow through the organs, increases reactive oxygen species production and contributes to endothelium damage [9]. This explains why the connections between EMPs and arterial stiffness are complex and multidirectional. Thus far, there have not been any studies assessing an endothelium state established by the EMPs level in the blood serum, and the aortic stiffness parameters assessed in echocardiography at the same time in patients with T2DM.

## 2. Aim of the Study

The aim of this study was to noninvasively assess endothelial function in patients with T2DM by measuring the EMPs level in the blood serum and the ascending aorta stiffness parameters in transthoracic echocardiography.

## 3. Material and Methods

The authors obtained consent for conducting this study from the Bioethics Committee of the Medical University of Lublin. The patients gave written informed consent to participate in the study after being presented with the study protocol.

### 3.1. Patients

In this study, 58 patients admitted to the Department of Internal Diseases were included. The studied group consisted of 38 patients with T2DM diagnosed earlier or during present hospitalization. The diagnosis of T2DM was based on WHO criteria [10]. The control group consisted of 20 patients without carbohydrate metabolism disorders (oral glucose tolerance test was conducted to exclude diabetes) and other possible concomitant diseases (heart arrhythmia, aortic valve defects, neoplasms, renal or liver insufficiency and acute or chronic inflammatory diseases). Obesity and hypertension (diagnosed according to European Society of Cardiology (ESC) criteria) [11] were not among the exclusion criteria.

### 3.2. Endothelium Assessment

The identification of EMPs was performed with flow cytometry [12,13]. The tests were performed on blood serum obtained from the patients after 12 h of fasting period and collected to tubes with a clot activator. The samples were stored at 20–25 °C during transport. The period of time from collecting the samples to the laboratory test did not exceed 2 h. The laboratory tests were performed using CD31-FITC (Beckman Coulter, Marseille, France) and CD42b-PE (Beckman Coulter, Marseille, France) antibodies. It was run with a Navios (Beckman Coulter, Brea, US) flow cytometer. The alignment and calibration checks were performed daily on the flow cytometer using FlowCheckPro. The EMPs were identified as CD31-positive and CD42b-negative particles with diameter <1 μm [14]. The amount of EMPs is presented as their amount in 1 μl of tested blood serum.

### 3.3. Aortic Stiffness Assessment

The ascending aorta stiffness was assessed during transthoracic echocardiography performed with a Vivid 4 device (GE Healthcare, Chicago, IL, USA) and 2.2 MHz convex transducer. Electrocardiography (ECG) was also performed during the examination. The maximal aortic diameter (Ao_max_) when the aortic valve was open and the minimal aortic diameter (Ao_min_) at the peak of R wave in ECG performed at the same time [15] were assessed in this study. Blood pressure was measured on the brachial artery using a proper cuff. The mean systolic (SBP) and diastolic (DBP) blood pressure, mean pulse pressure (PP) and mean arterial pressure (MAP) were calculated. The results mentioned above served to calculate the aortic stiffness parameters. The aortic stiffness index beta was calculated as a quotient of the natural logarithm of pressure alterations to the change of the vessel’s diameter, according to the equation [16,17,18]:

(1) $$\beta \;=\frac{\ln\;\left(\mathrm{SBP}/\mathrm{DBP}\right)}{\left({\mathrm{Ao}}_{\max}-{\mathrm{Ao}}_{\min}\right)/{\mathrm{Ao}}_{\min}}$$

Epsilon (Ep)—pressure-related vessel elasticity index, which shows the value of the blood pressure change necessary to increase the vessel diameter by 100%, was calculated as in equation below [16,17,18]:

(2) $$\mathrm{Ep}\;\left[\mathrm{mm}\;\mathrm{Hg}\right]\;=\frac{\left(\mathrm{SBP}\;-\;\mathrm{DBP}\right)}{\left({\mathrm{Ao}}_{\max}-{\mathrm{Ao}}_{\min}\right)/{\mathrm{Ao}}_{\min}}$$

The aortic compliance index D, which is reciprocal of epsilon, was calculated as in equation below [16]:

(3) $$D\;\left[{\mathrm{mm}\;\mathrm{Hg}}^{-1}\right]\;=\frac{{\mathrm{Ao}}_{\max}-{\mathrm{Ao}}_{\min}}{{\mathrm{Ao}}_{\min}\left(\mathrm{SBP}-\mathrm{DBP}\right)}$$

### 3.4. Biochemical Testing

The blood samples were drawn from the patients 12 h after their last meal. The following parameters were measured: The lipid profile (total cholesterol, high-density lipoprotein (HDL), triglycerides; the low-density lipoprotein (LDL) level was calculated in accordance with Friedewald’s equation [19]; creatinine with the estimated glomerular filtration rate (eGFR) was calculated with a simplified modification of diet in renal diseases (MDRD) formula (taking into account age, sex of the patients and level of creatinine) [20]; C-reactive protein (CRP), and glycated hemoglobin (Hb_A1c_) expressed as a percentage (%).

### 3.5. Statistical Analysis

The statistical analysis was performed using Statistica 10 PL computer software. The results are presented as the mean value ± standard deviation in the case of quantitative variables and as a percentage is the case of qualitative variables. The statistical significance was set for *p* < 0.05.

The studied parameters/quantitative variables convergence with a normal distribution was tested with Shapiro-Wilk test. The student’s t-test and ANOVA analysis of variance was used for the variables and groups of variables convergent with a normal distribution. When the variables distribution was significantly different from normal, the Mann-Whitney U test and one-way ANOVA on ranks (Kruskal-Wallis test) were applied. The correlations between the studied parameters were explored using the Pearson correlation coefficient or Spearman’s rank correlation coefficient for variables with a distribution different from normal.

## 4. Results

### 4.1. Overall Characteristics of Control and Studied Groups

The age of the patients in studied and the control group did not differ significantly (61.1 ± 9.8 years versus 61.7 ± 9.6 years; *p* = 0.840). Men constituted 42.1% of studied group and 40% of the control group and the difference was not statistically significant (*p* = 0.461). The high BMI values were observed both in the studied and control group (31.3 ± 5.0 kg/m^2^ versus 29.8 ± 4.1 kg/m^2^; *p* = 0.261). A majority of patients in both groups were hypertensive − 81.6% of the studied group and 80% of the control group (*p* = 0.895).

The SBP, DBP and MAP values did not significantly differ between the groups (respectively *p* = 0.209; *p* = 0.52; *p* = 0.723). There were no significant differences in the lipid profile, renal function parameters and CRP level between the studied and the control group (Table 1).

### 4.2. Aortic Stiffness Parameters and Endothelium State Assessment

The beta index was 3.52 ± 0.45 in the studied group and 3.30 ± 0.50 in the control group. The elasticity index (Ep) in patients with T2DM was 1160.0 ± 573.4 mm Hg versus 905.4 ± 501.2 mm Hg in the control group, this difference was not statistically significant (p = 0.094). The aortic compliance D was 1.03 ± 0.51 × 10−3 mm Hg−1 in the studied group and 1.45 ± 0.79 × 10^−3^ mm Hg^−1^ in the control group (*p* = 0.058). The level of EMPS was not significantly higher among patients with T2DM than among those in the control group (20.8 ± 24.7 versus 9.9 ± 7.5; *p* = 0.223).

### 4.3. Hypertension Coexisting with T2DM and Amount of EMPs and Aortic Stiffness Parameters

**Figure 1:**
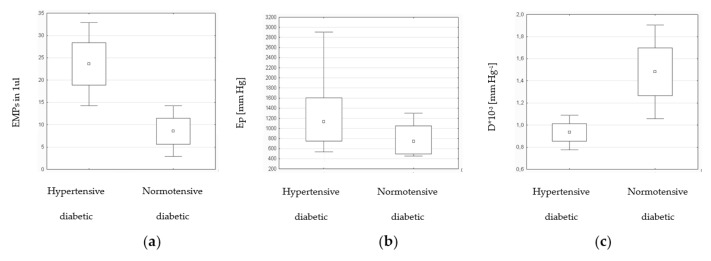
The number of EMPs (**a**) and aortic stiffness parameters: Ep (**b**), D (**c**) among patients with T2DM and hypertension in comparsion with normotensive diabetic patients. EMPs, endothelial microparticles; Ep, elasticity index; D, compliance index; T2DM, type 2 diabetes mellitus.

The patients with T2DM were divided into two subgroups (Table 2). The patients with diabetes and hypertension presented higher levels of EMPs in comparison with diabetic normotensive patients. This difference was statistically significant (Figure 1a). Among patients with T2DM and hypertension aortic stiffness assessed with Ep (Figure 1b) was higher and aortic compliance D (Figure 1c) was lower than in normotensive diabetic group.

### 4.4. Influence of Glycemic Control Assessed with Hb_A1c_ on EMPs Level and Aortic Stiffness Parameters

In order to perform further analysis, the studied group was divided into subgroups depending on glycemic control according to the Hb_A1c_. The EMPs level, β, Ep and D indexes did not significantly differ between the subgroups with Hb_A1c_ higher and lower than 8% (Table 3).

### 4.5. Correlations between EMPs Level, Aortic Stiffness Parameters and Arterial Pressure Values among Patients with T2DM

**Figure 2:**
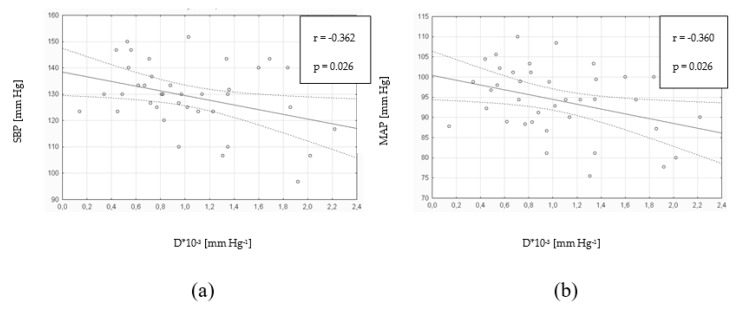
Aortic compliance D in relation to SBP (**a**) and MAP (**b**) among patients with T2DM (N = 38). D, compliance index; SBP, systolic blood pressure; MAP, mean arterial pressure; T2DM, type 2 diabetes mellitus.

There was a statistically significant correlation between the amount of EMPs and SBP (r = 0.355; *p* = 0.029; N = 38) and between the amount of EMPs and PP (r = 0.381; *p* = 0.018; *N* = 38). However, there was no correlation between the amount of EMPs and DBP (r = 0.081; *p* = 0.629; *N* = 38) nor between the EMPs level and MAP (r = 0.236; *p* = 0.154; *N* = 38). There were statistically significant positive correlations between the Ep index and DBP as well as MAP (Table 4). The aortic compliance D was negatively correlated with SBP and MAP (Figure 2).

### 4.6. Correlations between EMPs Level and Aortic Stiffness Parameters among Patients with T2DM

In the presented study, there were no statistically significant correlations observed between the EMPs level and β index (r = -0.059; *p* = 0.723; *N* = 38), Ep index (r = 0.061; *p* = 0.714; *N* = 38) and compliance D (r = 0.003; *p* = 0.985; *N* = 38) among patients with T2DM.

### 4.7. Correlations between EMPs Level, Aortic Stiffness Parameters and Biochemical Parameters among Patients with T2DM

There was no correlation found between the amount of EMPs and lipid profile components, CRP level and glycemia in the studied group. There was a statistically significant positive correlation between the creatinine level and the amount of EMPs (r = 0.411; *p* = 0.010; *N* = 38). There was also a significant negative correlation between the amount of EMPs and eGFR (r = -0.339; *p* = 0.037; N = 38). There were no statistically significant correlations between the aortic stiffness parameters and the lipid profile, renal function parameters, CRP level and glycemia in studied group.

## 5. Discussion

The assessment of early atherosclerotic lesions in the arteries would be useful in establishing cardiovascular risk in patients without clinical symptoms [21]. This is why noninvasive endothelium function markers in relation to aortic stiffness assessment in T2DM were evaluated in the presented study.

The level of EMPS was not significantly higher among patients with T2DM than among those in control group. Koga et al. reported a significantly higher amount of CD144+ EMPs in patients with T2DM in comparison with the control group. This study also suggested that increased EMPs level is an important cardiovascular risk factor [22]. However, the connection between the amount of EMPs and diabetes is not clear. Tsimerman et al. observed significantly increased EMPs level in patients with T2DM and its complications, including advanced diabetic foot ulcers and ischemic heart disease, in comparison with healthy individuals [23]. On the other hand, the patients with T2DM who had acute coronary syndrome from 6 weeks to 6 months before testing for the EMPs level, presented significantly lower CD31+/CD41- EMPs level than healthy individuals [24]. These results may indicate the possibility of the EMPs level decrease caused by intensive treatment after cardiovascular event in patients with diabetes. In another study, the treatment with pioglitazone in patients with metabolic syndrome was reported to cause a reduction of the CD31+/CD42b- EMPs level [25]. Considering the fact that patients included in this study were treated with hypoglycemic, hypolipidemic and hypotensive agents, a lack of a EMPs level correlation with T2DM could be explained by modifying amount of EMPs with multifactorial intervention. This study observed a significantly increased EMPs level in patients with T2DM coexisting with hypertension in comparison with normotensive diabetic patients. Furthermore, the amount of EMPs was positively correlated with SBP, PP and creatinine levels and negatively correlated with eGFR. Chen et al. presented similar results, in his study, the patients with hypertension and T2DM had higher CD31+/CD42- EMPs level in comparison with normotensive individuals [26]. The authors of this publication also found a correlation between the EMPs level and SBP and MAP. Except for the age and diabetes duration, the EMPs level turned out to be an independent risk factor of developing hypertension in diabetic patients [26]. Another study revealed a positive correlation between the amount of CD31+/CD42- as well as CD51+ EMPs and SBP in patients with T2DM [27]. Preston et al. in a study conducted on patients with severe hypertension observed a significant increase of CD31+/CD42- EMPs, which level was positively correlated with SBP, DBP and the presence of diabetes [28]. The EMPs level increase may not only be the endothelial damage marker, but it can also have a negative impact on the endothelium itself and lead to the development and progression of hypertension. Due to the combined effects of endothelial and platelet microparticles on coagulation, leukocytes, and endothelium, it is possible that they play a pathogenic role in mediating target organ injury in severe hypertension [28].

This study also observed that, except for hypertension, renal function disorders are connected with higher amount of EMPs, which may lead to the acceleration of atherosclerotic process in course of diabetes. Huang et al. found CD31+ EMPs level to be higher among hypertensive patients with albuminuria in comparison with hypertensive patients without this condition [28]. The authors of this study suggested that the increased EMPs level was connected with the decreased ability of the vessels to regenerate, which could accelerate atherosclerosis progression and increase cardiovascular risk in hypertensive patients with nephropathy [29]. Trappenburg et al. observed significant increase of the CD144+ EMPs level in patients with chronic kidney disease and in hemodialysis patients in comparison with healthy individuals. The authors of this work suggest that these results may illustrate endothelial damage by chronic exposure to uremic toxins in blood and/or concomitant atherosclerosis [30]. A prospective study conducted on hemodialysis patients showed that the amount of CD31+/CD41- EMPs is a strong and independent predictive factor of death and cardiovascular death [31]. Thus, the amount of EMPs may become an independent, noninvasive marker of endothelial dysfunction, but it may as well be useful in diabetic nephropathy risk assessment.

In this study, hypertensive patients with T2DM presented significantly increased aortic stiffness measured with the Ep index and significantly decreased aortic compliance D in comparison with normotensive diabetic patients. Furthermore, moderate positive correlations were found between the Ep index and SBP, DBP and MAP as well as significant negative correlations between the aortic compliance D and SBP, MAP. Zapolski et al. obtained similar results in their study patients with T2DM had increased aortic stiffness β assessed with echocardiography in comparison with healthy individuals [32]. In another study performed on patients with metabolic syndrome, aortic compliance was significantly lower in hypertensive patients than in normotensives [33]. Avgeropoulou et al. drew similar conclusions by demonstrating the correlation of SBP and the stiffness index β and Ep index in diabetic patients [34]. It is worth noticing that hypertension coexisting with T2DM is an additional risk factor of arterial stiffening and leads to the acceleration of atherosclerosis development. Arterial stiffness may constitute a long-term cardiovascular risk factor due to the fact that arterial stiffness reduction requires maintenance of biochemical disorders correction obtained in the treatment process [5].

This study did not find any significant correlation between the amount of EMPs and aortic stiffness parameters. These results are contradictory to some other research results. Wang et al. observed that increased arterial stiffness assessed with brachial-ankle PWV is positively correlated with CD31+/CD42- EMPs level in healthy individuals [35]. Similar results were obtained in patients with T2DM. Higher CD31+/CD42- i CD51+ EMPs level was found in diabetic patients, and the amount of EMPs was positively correlated with brachial-ankle PWV [28]. On the other hand, Kinouchi et al. presented increased brachial-ankle PWV values in diabetic patients, yet this result did not correlate with endothelial dysfunction measured with the extensibility of brachial artery [36]. This corresponds to our research, in which no correlation was found between arterial stiffness and endothelial dysfunction assessed with the EMPs level. The incoherence of various studies results may be an effect of heterogeneous methods of identifying EMPs and using different means of arterial stiffness assessment. The lack of significant correlations in the patients may be due to the fact that the EMPs level and aortic stiffness parameters are used to assess various aspects of atherosclerotic process and the pieces of information they bring are independent from each other. The results obtained in this study seem to be supporting the hypothesis that endothelial damage measured with the EMPs level is caused mostly by hypertension and indirectly, by an arterial stiffening process in the course of T2DM.

The authors of the presented study are aware of its limitations. The research was conducted on a small number of patients and only in one clinical center. The lack of standardized EMPs detection methods among publications on this subject constituted a significant limitation to this work. Thus, the EMPs level results are difficult to compare with other studies. Taking into consideration the fact that amount of CD31+/CD42b- and CD62E+ EMPs rises in −80 °C temperature [13], the EMPs were detected in non-frozen plasma. In addition, arterial stiffness was assessed only within the ascending aorta, whereas it may differ in other vessels. It is worth noticing that the ascending aorta diameter was measured while blood pressure was measured on the peripheral artery. This may lead to an overestimation of the blood pressure value. Some authors consider this methodological aspect as irrelevant [15].

## 6. Conclusions

In conclusion, this study observed that hypertension coexisting with T2DM had a negative impact on the endothelium state assessed with the EMPs level. In addition, the EMPs level in diabetic patients increased along with renal failure progression. Hypertension coexisting with T2DM was also connected with increased aortic stiffness. In contrast, this study did not find any correlation of the lipid profile, CRP level and glycemic control measured with Hb_A1c_ with aortic stiffness parameters and the EMPs level. Furthermore, this study did not find any significant correlation between the amount of EMPs and the aortic stiffness parameters. Thus, the EMPs level in patients with T2DM seems to constitute information complementary but not identical to aortic stiffness parameters. Considering the elevated number of EMPs in diabetic patients with hypertension as well as the positive correlation between EMPs and serum creatinine level, EMPs assessment could be useful in selecting patients at high risk of organ damage in diabetes. Additional investigations in this field are necessary to fully define its effect.

## Figures and Tables

**Table 1:** Laboratory parameters in the study and control group (mean ± standard deviation).

	Study Group (N = 38)	Control Group (N = 20)	*P*-Value
Total cholesterol [mg/dL]	187.5 ± 36.8	192.1 ± 49.0	0.691
LDL [mg/dL]	121.9 ± 68.3	130.1 ± 56.5	0.299
HDL [mg/dL]	50.9 ± 22.2	48.7 ± 18.3	0.883
Trigliceride [mg/dL]	163.5 ± 103.7	123.9 ± 57.1	0.174
Creatinine [mg/dL]	0.87 ± 0.26	0.81 ± 0.14	0.793
eGFR [mL/min/ 1,73 m^2^]	88.7 ± 28.3	89.4 ± 16.8	0.911
CRP [mg/L]	3.93 ± 3.21	2.49 ± 1.51	0.159

LDL, low-density lipoprotein cholesterol; HDL, high-density lipoprotein cholesterol; eGFR estimated glomerular filtration rate; CRP, C-reactive protein.

**Table 2:** EMPs level and aortic stiffness parameters among hypertensive and normotensive patients with T2DM (mean ± standard deviation).

	Study Group with Hypertension (N = 31)	Study Group without Hypertension (N = 7)	*P*-Value
EMPs	23.6 ± 26.4	8.6 ± 7.7	0.011 *
β	3.57 ± 0.46	3.31 ± 0.33	0.159
Ep [mm Hg]	1246.6 ± 585.6	776.4 ± 319.7	0.032 *
D × 10^−3^ [mm Hg^−1^]	0.93 ± 0.45	1.48 ± 0.57	0.008 *

EMPs, endothelial microparticles; β, stiffness index; Ep, elasticity index; D, compliance index; T2DM, type 2 diabetes mellitus. * Statistically significant.

**Table 3:** EMPs level and aortic stiffness parameters in study group divided by the level of glycemic control measured by Hb_A1c_ (mean ± standard deviation).

	Study Group Hb_A1c_<8% (N = 20)	Study Group Hb_A1c_>8% (N = 18)	*P*-Value
EMPs	23.3 ± 28.3	18.1 ± 20.5	0.573
β	3.61 ± 0.51	3.43 ± 0.35	0.239
Ep [mm Hg]	1307.1 ± 657.9	996.4 ± 421.8	0.096
D x 10^−3^ [mm Hg^−1^]	0.90 ± 0.48	1.19 ± 0.51	0.081

EMPs, endothelial microparticles; β, stiffness index; Ep, elasticity index; D, compliance index; Hb_A1c_, glycated hemoglobin.

**Table 4:** Aortic stiffness parameters in relation to parameters of blood pressure among patients with T2DM (N = 38).

	β		Ep [mm Hg]		D × 10^−3^ [mm Hg^−1^]	
	R-Value	*P*-Value	R-Value	*P*-Value	R-Value	*P*-Value
SBP [mm Hg]	0.155	0.353	0.369	0.023 *	−0.362	0.026 *
DBP [mm Hg]	0.291	0.760	0.367	0.024 *	−0.294	0.740
MAP [mm Hg]	0.255	0.123	0.403	0.012 *	−0.360	0.026 *
PP [mm Hg]	−0.030	0.857	0.237	0.153	−0.232	0.161

SBP, systolic blood pressure; DBP, diastolic blood pressure; MAP, mean arterial pressure; PP, pulse pressure; β, stiffness index; Ep, elasticity index; D, compliance index; T2DM, type 2 diabetes mellitus. * Statistically significant.
